# A physics-informed neural SDE network for learning cellular dynamics from time-series scRNA-seq data

**DOI:** 10.1093/bioinformatics/btae400

**Published:** 2024-09-04

**Authors:** Qi Jiang, Lin Wan

**Affiliations:** Academy of Mathematics and Systems Science, Chinese Academy of Sciences, Beijing 100190, China; School of Mathematical Sciences, University of Chinese Academy of Sciences, Beijing 100049, China; Academy of Mathematics and Systems Science, Chinese Academy of Sciences, Beijing 100190, China; School of Mathematical Sciences, University of Chinese Academy of Sciences, Beijing 100049, China

## Abstract

**Motivation:** Learning cellular dynamics through reconstruction of the underlying cellular potential energy landscape (aka Waddington landscape) from time-series single-cell RNA sequencing (scRNA-seq) data is a current challenge. Prevailing data-driven computational methods can be hampered by the lack of physical principles to guide learning from complex data, resulting in reduced prediction accuracy and interpretability when applied to infer cell population dynamics.

**Results:** Here, we propose PI-SDE, a physics-informed neural stochastic differential equation (SDE) framework that combines the Hamilton–Jacobi (HJ) equation and neural SDE to learn cellular dynamics. Grounded in potential energy theory of biological systems, PI-SDE integrates the principle of least action by enforcing the HJ equation when reconstructing cellular potential energy function. This approach not only facilitates accurate predictions, but also improves interpretability, especially in the reconstructed potential energy landscape. Through benchmarking on two real scRNA-seq datasets, we demonstrate the importance of incorporating the HJ regularization term in dynamic inference, especially in predicting gene expression at held-out time points. Meanwhile, the learned potential energy landscape provides biologically interpretable insights into the process of cell differentiation. Our framework enhances model performance, while maintaining robustness and stability.

**Availability:** PI-SDE software is available at https://github.com/QiJiang-QJ/PI-SDE.

## 1 Introduction

The dynamics of cellular developmental processes (e.g. cell differentiation and tumorigenesis) are highly complex. Emerging time-series single-cell RNA sequencing (scRNA-seq) data allow single cell-resolved study of heterogeneous cell populations, providing a systematic approach to reveal underlying developmental dynamics, cell–cell communication, and gene regulatory networks. However, scRNA-seq techniques can destroy cells during sequencing, resulting in a lack of direct correspondence between cells collected at different time points. The analysis of time series scRNA-seq data could be comprised of (i) distortion created by assorted sources of data collection and generation across time samples and (ii) inheritance of cell-to-cell variations by stochastic and nonlinear dynamic patterns of gene expression. Thus, reconstruction of the underlying cellular dynamics from the unpaired time series data remains challenging.

Waddington’s epigenetic landscape, a concept of embryonic development, is a classic metaphor for cellular differentiation in biology (Waddington 1957). It illustrates various developmental trajectories a cell might take toward differentiation as balls rolling down branching valleys. A growing number of computational methods are being developed to reconstruct the cellular developmental landscape (also known as the Waddington landscape) based on time series scRNA-seq. Among the existing methods, Waddington-optimal transport (OT) reports groundbreaking work that develops an unbalanced OT framework to infer probabilistic couplings between consecutive measurements (Schiebinger *et al.* 2019). Extending the OT framework, Yang and Uhler (2019) utilize a generative adversarial framework to directly parameterize the OT map. However, these methods learn static and linear mappings between irregularly sampled time points, limiting the capacity of the model to fully capture the continuous and nonlinear nature of cellular dynamics.

To address these limitations, dynamical model-based machine learning methods have recently been developed to characterize the continuous, nonlinear, and even stochastic nature of cellular dynamics. For example, TrajectoryNet combines dynamic OT and continuous normalized flows to learn the optimal flow of evolving cell populations with neural ordinary differential equations (Tong *et al.* 2020). To account for the stochasticity of cellular dynamics, PRESCIENT models cellular differentiation using stochastic differential equations (SDEs) in which a deterministic part, or “drift” term, is defined as the negative gradient of a potential function. PRESCIENT uses a neural network to decode the underlying landscape of cellular development, opting to learn this energy potential function, rather than directly modeling the optimal flow (Yeo *et al.* 2021). MIOFlow builds on TrajectoryNet by incorporating SDEs, along with integrating a geodesic autoencoder to further extend dynamic OT into latent space (Huguet *et al.* 2022).

Despite these advances, the aforementioned computational methods can still struggle when analyzing time-series scRNA-seq data. For example, the performance of these data-driven methods can degrade significantly under distribution shifts, also known as out-of-distribution (OOD), e.g. when the probability distribution changes between training and test data. This situation frequently arises in dynamic modeling, particularly when predicting data at unknown time points. This vulnerability is exacerbated by over-reliance on the training data, leading to overfitting and a subsequent decline in the ability of the models to generalize to new, unseen data (Oh *et al.* 2024). In addition, these data-driven methods often treat the complex systems as “black boxes” and, consequently, ignore the underlying physical principles that generate the data, in turn resulting in a lack of interpretability. These limitations call for improvements in both prediction accuracy and interpretability in the analysis of time-series scRNA-seq.

Here, we propose PI-SDE, a physics-informed neural SDE network framework designed to learn the cellular dynamics and underlying potential energy landscape from time-series scRNA-seq data. PI-SDE extends the SDE-based framework of the PRESCIENT method by incorporating the principle of least action. The principle of least action is a fundamental concept in both physics and biology, which states that the optimal path taken by a system between two states is the one for which the action is minimized (Goldstein 2011, Fang *et al.* 2019). Recent advances in energy landscape theory provide a conceptual framework for analyzing the global stability and transition dynamics in biological systems (Wang 2015). In particular, the defined potential energy function, also called the quasipotential, becomes a powerful analytical tool for calculating the minimum action path of the noise-induced transition in multistable systems (Wang 2015, Bressloff 2021). Large deviation theory provides a rigorous foundation that the quasipotential satisfies the Hamilton–Jacobi (HJ) equation within the context of SDEs with weak noise (Bressloff 2021, Weinan *et al.* 2021). The HJ equation motivates an important characterization for the least action path. Leveraging the conceptual framework of quasipotential, we develop a physics-informed loss function that integrates the HJ regularization term to penalize violations of HJ equations in our learning of the SDE neural network. The use of our physics-informed loss function ensures both robustness and predictive accuracy of the model in the presence of OOD; it also ensures that the learned potential energy function is governed by the physical principles of least action embodied in the HJ equation. Thus, PI-SDE improves both prediction accuracy and biological interpretability by harnessing the power of physical laws and neural networks.

We benchmark the performance of PI-SDE on two time-series scRNA-seq datasets, including pancreatic *β*-cell differentiation (Veres *et al.* 2019) and mouse hematopoiesis (Weinreb *et al.* 2020). We compare the performance of PI-SDE with current state-of-the-art time-series scRNA-seq inference methods, namely TrajectoryNet, PRESCIENT, and MIOFlow. We demonstrate that (i) PI-SDE achieves superior performance compared to that of TrajectoryNet, PRESCIENT, or MIOFlow in predicting unseen time points; (ii) PI-SDE reconstructs an interpretable potential energy landscape that quantifies cell differentiation potency, in closer alignment with the original concept of the Waddington landscape, where cells with higher potential energy tend to transition to the lower potential energy states; and (iii) PI-SDE stabilizes the training process and achieves robust performance, especially when noise is introduced. Overall, our study highlights the critical role of embedding physical principles in computational models, paving the way for a deeper understanding of the complex biological phenomena.

## 2 Materials and methods

### 2.1 Overview of PI-SDE

**Figure 1.**
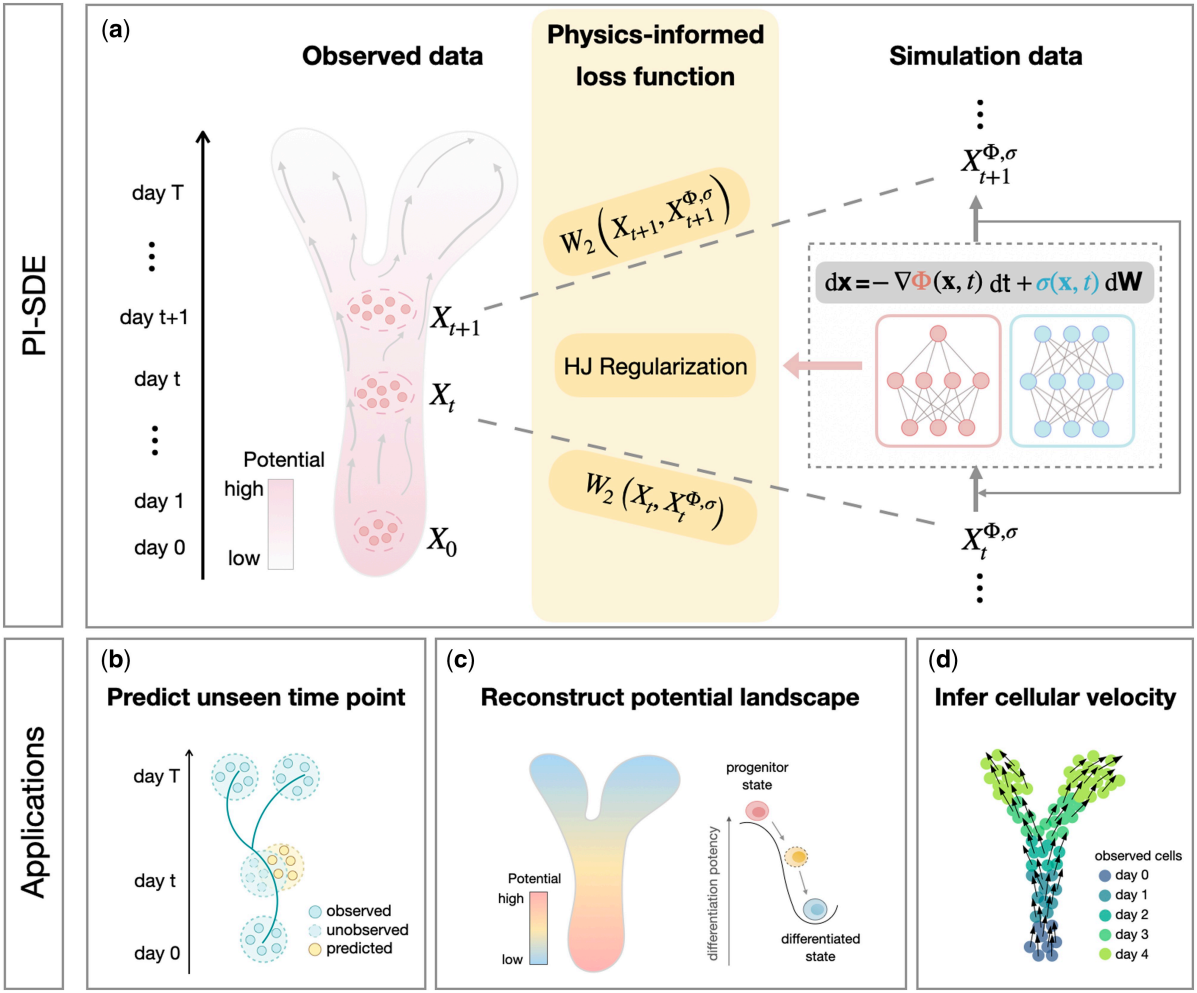
Overview of PI-SDE. (a) PI-SDE combines neural SDEs and physics-informed insights to learn cellular dynamics from time-series scRNA-seq data. Specifically, we depict cellular differentiation as diffusion processes governed by SDEs with both drift and diffusion coefficients modeled through separate neural networks. By integrating HJ regularization, the resulting physics-informed loss function guides our model to find the optimal potential landscape with accurate prediction and biological interpretability. PI-SDE takes the input of time-series scRNA-seq data and outputs (b) the predicted unseen data, (c) the reconstructed energy potential landscape that resembles the original Waddington landscape, and (d) the inferred cellular velocity.

PI-SDE extends the framework of PRESCIENT by combining the physics-informed principle to infer the complex dynamics of cellular processes from time-series scRNA-seq data (Fig. 1a). It models cellular differentiation as a diffusion process, as described by the SDE, whereby the drift term of the SDE is expressed as the negative gradient of a potential function that drives the cellular dynamics. PI-SDE also enforces potential function to satisfy HJ equations, proven to be an essential property of the quasipotential (Wang 2015, Fang *et al.* 2019, Bressloff 2021), by incorporating an HJ regularization term into the loss function. PI-SDE parameterizes potential function and diffusion coefficient by two separate neural networks and learns the parameters under the Neural SDE framework (Li *et al.* 2020, Kidger *et al.* 2021). PI-SDE takes the input of time-series scRNA-seq data and outputs (i) predicted gene expressions at unseen time points (Fig. 1b), (ii) the reconstructed potential energy landscape that resembles the original Waddington landscape (Fig. 1c), and (iii) inferred cellular velocity (Fig. 1d).

### 2.2 Notation

Suppose time series single-cell samples are collected at (T+1) time points given by:

(1) $$\left(t_{0},X_{t_{0}}\right),\left(t_{1},X_{t_{1}}\right),\ldots ,\left(t_{T},X_{t_{T}}\right),$$

where $X_{t_{l}}={\left\{x_{t_{l},i}\right\}}_{i=1}^{N^{l}}\in R^{N^{l}\times d}$ is a set of cells at *d*-dimensional space, either in the original gene expression space or the low-dimensional space obtained from dimension reduction at time $t_{l}(l=0,1,\ldots ,T)$.

At each observed time *t_l_*, cells are assumed to be sampled from a probability distribution. The resulting empirical distribution of $X_{t_{l}}$ is represented by ${\hat{\rho }}_{t_{l}}$. Our goal is to find a stochastic process, accompanied by a time-varying probability distribution $\left\{x_{t}∼\rho_{t}:t_{0}\leq t\leq t_{T}\right\}$, capable of connecting the distributions $\left\{{\hat{\rho }}_{t_{l}}:l=0,1,\ldots ,T\right\}$ at all observed times.

### 2.3 Mathematical formulation of PI-SDE

We model the cellular dynamics as a diffusion process $\left\{x_{t}∼\rho_{t}:t_{0}\leq t\leq t_{T}\right\}$, which is given by the following SDE:

(2) $$dx_{t}=f\left(x_{t},t\right)dt+\sigma \left(x_{t},t\right)dW_{t},\;t\in [t_{0},t_{T}].$$

Here, $f\left(x_{t},t\right)$ is the drift term that describes the changing trend of the process, $\sigma \left(x_{t},t\right)$ is the diffusion term that captures random fluctuations, and $W_{t}$ denotes standard Brownian motion. This SDE indicates that within a small time interval *dt*, the process $x_{t}$ changes as a result of both deterministic drift and random diffusion.

In practice, the diffusion coefficient $\sigma \left(x_{t},t\right)$ can be specified as a constant or as a function of $\left(x_{t},t\right)$. For the constant setting, σ may either be a predefined hyperparameter or a parameter optimized during training.

Our goal is to find a diffusion process $\left\{x_{t}∼\rho_{t}:t_{0}\leq t\leq t_{T}\right\}$ which approximates the distributions $\left\{{\hat{\rho }}_{t_{l}}:l=0,1,\ldots ,T\right\}$ at all observed times as closely as possible. To assess whether the learned process is close to the observed data, Wasserstein distance is employed to measure the discrepancy between empirical and predicted populations at observed time points as:

(3) $$\sum_{l=1}^{T}W_{2}{\left({\hat{\rho }}_{t_{l}},\rho_{t}\right)}^{2},$$

where $W_{2}{\left(\mu ,\nu \right)}^{2}$ is the 2-Wasserstein distance between two distributions *μ* and *ν*.

We further assume that cellular dynamics are governed by potential function, which quantifies the potency of cell differentiation. This potential function delineates the underlying cellular developmental landscape, showing the transition of cells from states of higher potential to those of lower potential.

We define the drift term as the negative gradient of a potential function. That is to say, a potential function $\Phi :R^{d}\times [t_{0},t_{T}]\to R$ exists such that:

(4) $$f\left(x_{t},t\right)=-\nabla_{x}\Phi (x_{t},t).$$

Since the solution of HJ equation corresponds to minimum action paths for the stochastic system, we therefore impose a constraint by which the potential function, or quasipotential, satisfies the HJ equation (Bressloff 2021). Here, in this study, we adopt the reduced form of HJ equation derived by Ruthotto *et al.* (2020) and Onken *et al.* (2021) as follows:

(5) $$-\partial_{t}\Phi (x,t)+\frac{1}{2}||\nabla_{x}\Phi (x,t)|{|}^{2}=0.$$

However, directly solving the optimization problem with the HJ constraint is a daunting task, so we relax the constraint of potential energy function in Equation (5) by introducing a regularization term which penalizes the violations of HJ equation along the trajectories as:

(6) $$R_{\text{HJ}}=\int_{t_{0}}^{t_{T}}\int_{R^{d}}\left|\partial_{t}\Phi (x,t)-\frac{1}{2}||\nabla_{x}\Phi (x,t)|{|}^{2}\right|\rho_{t}(x)dxdt.$$

Then, we integrate the HJ regularization to Equation (3) to derive a physics-informed loss function as:

(7) $$L_{\text{PI}}=\sum_{l=1}^{T}W_{2}{\left({\hat{\rho }}_{t_{l}},\rho_{t_{l}}\right)}^{2}+\lambda R_{\text{HJ}},$$

where *λ* is a hyperparameter determining the strength of the physics-informed term. This approach has already demonstrated its efficiency in addressing mean-field control problems (Ruthotto *et al.* 2020).

Note that PRESCIENT introduces an empirical regularization term to ensure that the potential energy function remains minimized at the final time point (Yeo *et al.* 2021). Nonetheless, this strategy raises critical issues concerning overfitting and generalizability, especially for long-term dynamic modeling. This is due to the fact that constraint at the final time point alone is insufficient, as the potential is not constrained at intermediate time points.

### 2.4 Model implementation and optimization

We formulate the inference of {Φ,σ} as the following dynamic optimization problem:

(8) $$\begin{array}{cc} \underset{\left\{\Phi ,\sigma \right\}}{\min}L_{\text{PI}} & =\sum_{l=1}^{T}W_{2}{\left({\hat{\rho }}_{t_{l}},\rho_{t_{l}}\right)}^{2}+\lambda E_{x_{t_{0}}∼{\hat{\rho }}_{t_{0}}}r(x_{t_{0}},t_{T}),\\ s.t.\;\;\;\;\; & \\ \partial_{t}\left(\begin{array}{c} x(x_{t_{0}},t)\\ r(x_{t_{0}},t) \end{array}\right) & =\left(\begin{array}{c} -\nabla_{x}\Phi (x(x_{t_{0}},t),t)dt+\sigma \left(x(x_{t_{0}},t),t\right)dW_{t}\\ \left|\partial_{t}\Phi (x(x_{t_{0}},t),t)-\frac{1}{2}||\nabla_{x}\Phi (x(x_{t_{0}},t),t)|{|}^{2}\right|dt \end{array}\right),\\ \left(\begin{array}{c} x(x_{t_{0}},0)\\ r(x_{t_{0}},0) \end{array}\right) & =\left(\begin{array}{c} x_{t_{0}}\\ 0 \end{array}\right),\\ x_{t_{0}}∼{\hat{\rho }}_{t_{0}}, & \;\rho_{t_{l}}=\text{Law}(x_{t_{l}}). \end{array}$$

Here, $x(x_{t_{0}},t)$ represents the state at time *t*, when $x_{t_{0}}$ is the initial state at time *t*_0_, $\text{Law}(x_{t_{l}})$ represents the predicted probability distribution of $x_{t_{l}}$ by the SDE given {Φ,σ}. Given an initial state $x_{t_{0}},\;r(x_{t_{0}},t)$ ($t_{0}\leq t\leq t_{T})$ is defined as:

(9) $$\int_{t_{0}}^{t}\left|\partial_{s}\Phi \left(x(x_{t_{0}},s),s\right)-\frac{1}{2}||\nabla_{x}\Phi \left(x(x_{t_{0}},s),s\right)|{|}^{2}\right|ds.$$

See Supplementary Note S2 for details.

This form allows us to compute the states of $x(x_{t_{0}},t)$ and $r(x_{t_{0}},t)$ for future times in parallel during the training process, thus significantly reducing computation time. Since it is intractable to traverse the entire function space, we parameterize {Φ,σ} through separate neural networks denoted as $\left\{\Phi_{\theta },\sigma_{\psi }\right\}$. Optimization details can be found in Supplementary Note S4. All models were trained using a single NVIDIA Tesla T4 GPU. We reported the computational time of PI-SDE and other baseline methods in Supplementary Table S1.

## 3 Results

We benchmarked PI-SDE against three state-of-the-art methods, TrajectoryNet, PRESCIENT (with or without considering growth rate), and MIOFlow, on two time-series scRNA-seq datasets, including pancreatic *β*-cell differentiation (Veres *et al.* 2019) (hereinafter denoted as Veres data) and mouse hematopoiesis (Weinreb *et al.* 2020) (hereinafter denoted as MH data).

**Figure 2.**
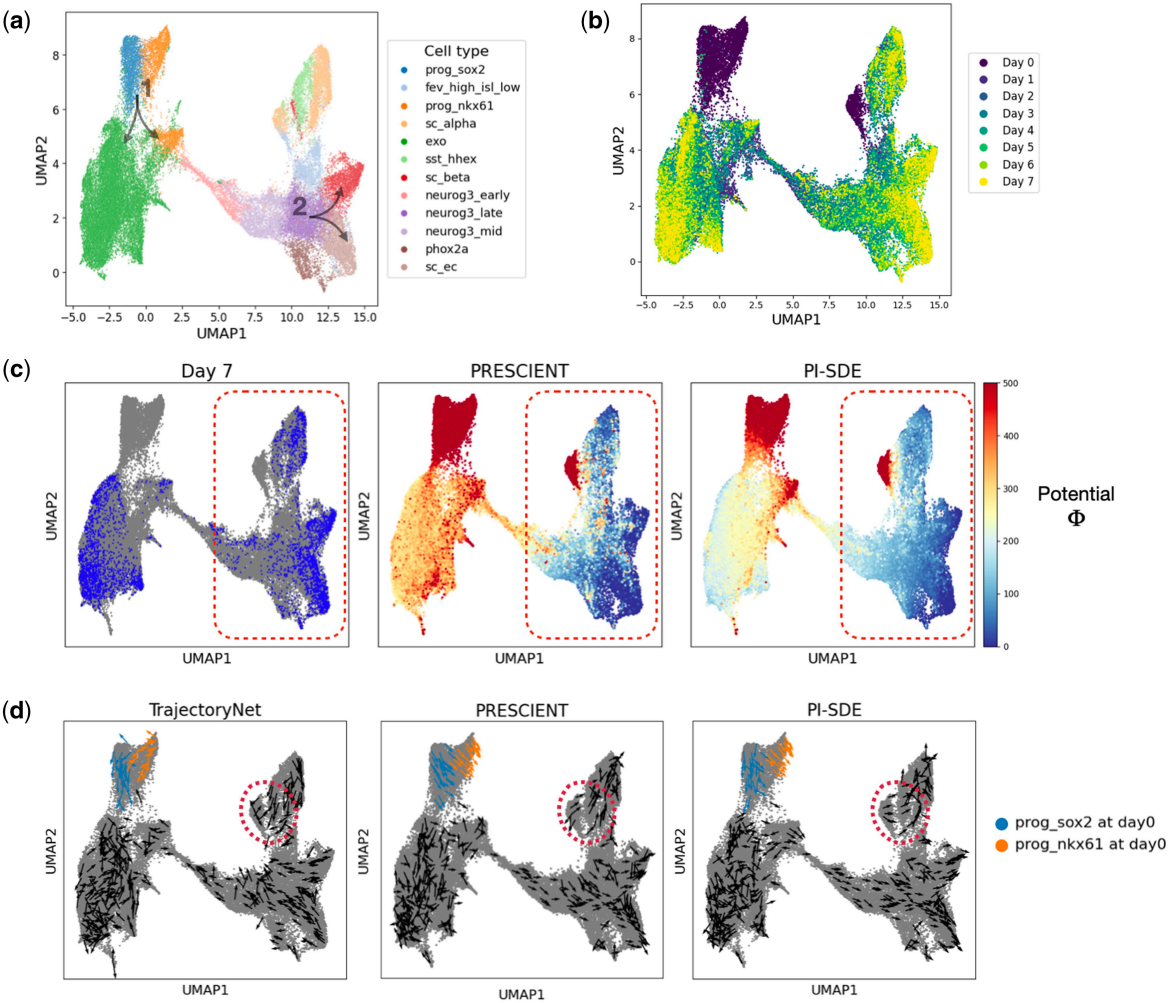
Reconstruction of potential energy landscape in pancreatic *β*-cell differentiation. (a) UMAP projections of cells sampled from stage 5 of pancreatic *β*-cell differentiation. Cells are colored according to their assigned cluster. Arrows indicate two key bifurcations. (b) UMAP plot projections of cells colored by time points. (c) Visualization of cells at final time point (left), potential energy landscape learned by PRESCIENT (middle) and PI-SDE (right). (d) Velocity for a random sample of observed cells inferred by TrajectoryNet (left), PRESCIENT (middle), and PI-SDE (right).

**Figure 3.**
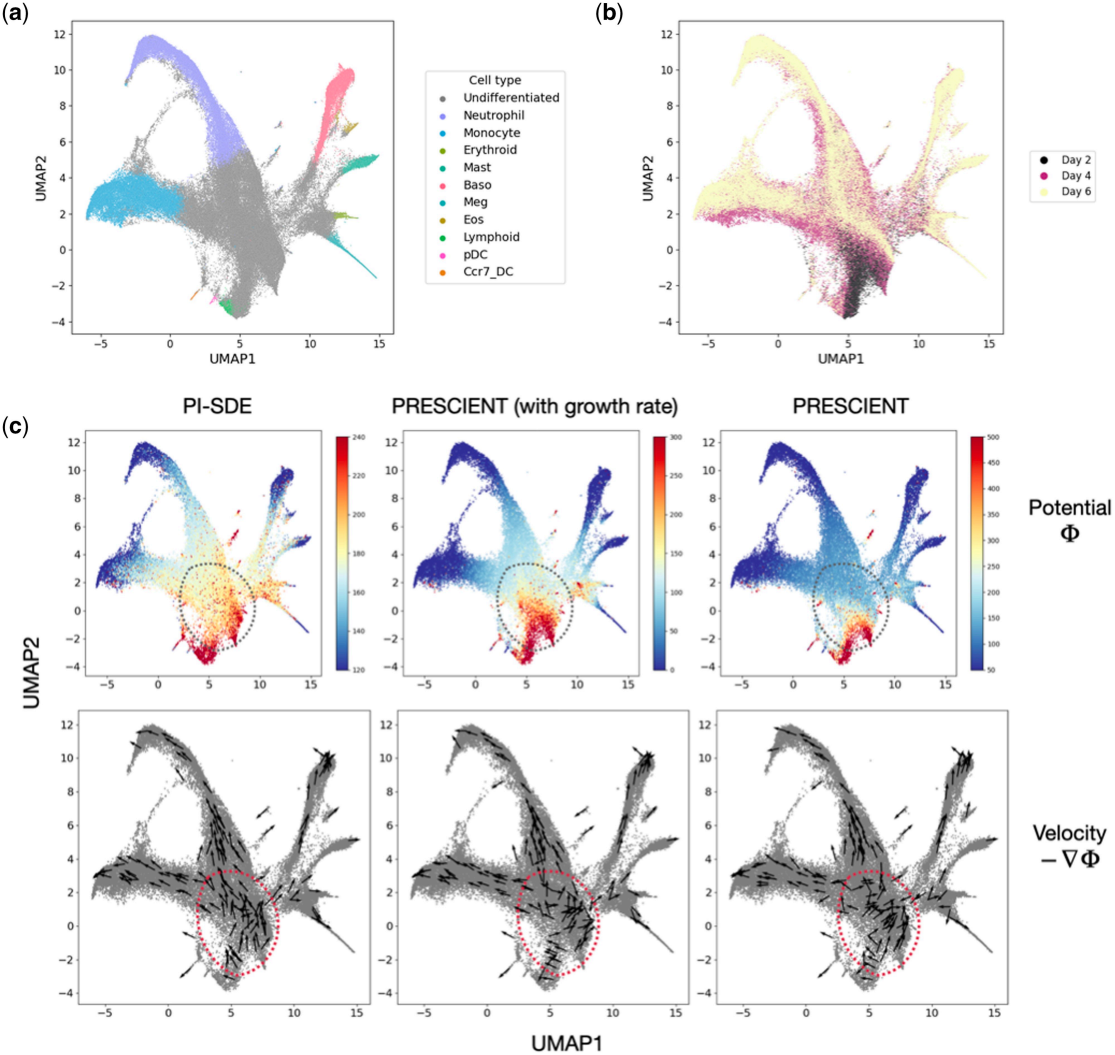
Reconstruction of potential energy landscape in mouse hematopoiesis. (a) UMAP projections of cells sampled in mouse hematopoiesis. Cells are colored according to their assigned cluster. (b) UMAP plot projections of cells colored by time points. (c) Visualization of potential energy landscape and velocity learned by PI-SDE (left), PRESCIENT with estimated growth rate (middle), and PRESCIENT without estimated growth rate (right).

Veres data comprise a comprehensive transcriptomic profile of 51 274 cells classified into 12 unique cell types. The Veres data span from Day 0 to Day 7, providing a detailed view of stage 5 pancreatic *β*-cell differentiation (Fig. 2a and b). MH data employ DNA barcodes to trace clonal trajectories throughout mouse hematopoiesis. Collected on days 2, 4, and 6, it comprises 130 887 cells across 11 cell types, including 49 302 cells with lineage tracing information (Fig. 3a and b) (Supplementary Note S1).

### 3.1 PI-SDE accurately predicts gene expressions at unseen time points

We first demonstrated the ability of PI-SDE to predict gene expressions at unseen time points by conducting held-out tasks on Veres data, where data from one (held-one-out) or multiple (held-multi-out) time points were excluded during training (Supplementary Note S6). After training, the accuracy of PI-SDE in predicting gene expression was compared with that of TrajectoryNet, PRESCIENT, and MIOFlow by assessing their performance at the excluded time points.

We assessed the performance of each model using Wasserstein distance, comparing results across five seeds of observed (training) and held-out (test) data (Supplementary Note S5). Overall, PI-SDE achieved the best average performance between the true and predicted gene expression profiles in terms of Wasserstein distance on Veres data across seven held-one-out tasks and three held-multi-out tasks. The second-best comparison approach is PRESCIENT (Tables 1 and 2; Supplementary Fig. S1 and Table S2).

**Table 1.** Held-one-out performance across five seeds on Veres data.

	Held-out *t* = 1		Held-out *t* = 2		Held-out *t* = 3		Held-out *t* = 4	
Model	Train	Test	Train	Test	Train	Test	Train	Test
TrajectoryNet	10.59 ± 1.08	12.81 ± 0.08	10.71 ± 1.01	11.69 ± 0.24	11.51 ± 0.49	9.62 ± 0.14	11.09 ± 0.83	10.38 ± 0.13
MIOFlow	10.09 ± 0.50	10.91 ± 0.02	10.33 ± 0.47	10.98 ± 0.22	10.37 ± 0.35	9.31 ± 0.18	10.34 ± 0.51	10.31 ± 0.11
PRESCIENT(+g)^a^	10.18 ± 1.19	11.45 ± 0.04	9.86 ± 1.16	9.54 ± 0.09	10.46 ± 1.10	8.11 ± 0.06	9.93 ± 1.08	9.06 ± 0.09
PRESCIENT	7.83 ± 0.37	10.45 ± 0.11	7.96 ± 0.37	8.91 ± 0.02	8.22 ± 0.37	7.52 ± 0.08	8.17 ± 0.34	7.79 ± 0.09
**PI-SDE**	**7.36 ± 0.32∗**	**10.36 ± 0.05**	**7.36 ± 0.40**	**8.35 ± 0.12**	**7.65 ± 0.39**	**7.41 ± 0.03**	**7.69 ± 0.44**	**7.61 ± 0.35**

The table presents results from four distinct held-out tasks, where Day 1, Day 2, Day 3, and Day 4 were excluded from the training process, respectively. For each task, we compute the average Wasserstein distance between observed data and predicted data (training loss) and Wasserstein distance between unseen data and predicted data (test loss).

* The bold values imply the best performance.

a PRESCIENT with estimated growth rate.

**Table 2.** Held-one-out performance across five seeds on Veres data (continued).

	Held-out *t* = 5		Held-out *t* = 6		Held-out *t* = 7	
Model	Train	Test	Train	Test	Train	Test
TrajectoryNet	11.06 ± 0.97	11.33 ± 0.08	10.99 ± 1.09	11.39 ± 0.16	10.76 ± 0.94	13.06 ± 0.45
MIOFlow	10.19 ± 0.53	10.59 ± 0.14	10.35 ± 0.52	10.68 ± 0.07	10.26 ± 0.59	11.01 ± 0.07
PRESCIENT(+g)^a^	9.77 ± 1.12	9.76 ± 0.36	9.76 ± 1.08	11.13 ± 0.20	9.85 ± 1.18	13.43 ± 0.19
PRESCIENT	8.08 ± 0.32	7.87 ± 0.10	8.10 ± 0.40	8.27 ± 0.14	7.92 ± 0.41	9.19 ± 0.04
**PI-SDE**	**7.51 ± 0.31** ∗	**7.40 ± 0.17**	**7.43 ± 0.30**	**7.66 ± 0.15**	**7.34 ± 0.33**	**8.61 ± 0.17**

The table presents results from three distinct held-out tasks where Day 5, Day 6, and Day 7 were excluded from the training process, respectively. For each task, we compute the average Wasserstein distance between observed data and predicted data (training loss) and Wasserstein distance between unseen data and predicted data (test loss).

* The bold values imply the best performance.

a PRESCIENT with estimated growth rate.

Tables 1 and 2 detailed the performance for each held-one-out task. In all subproblems, PI-SDE not only achieved the best fit on the training data, but also showed superior predictive ability on the test data, particularly excelling at predicting later time points. For instance, when Day 6 was excluded, PRESCIENT achieved a mean loss of 8.27 (without estimated growth rate), outperforming MIOFlow (10.68) and TrajectoryNet (11.39). PI-SDE achieved a test loss of 7.66, marking a 7.6% improvement over that of PRESCIENT and showcasing its long-term prediction accuracy. Additionally, PI-SDE clearly outperformed the baselines in three held-multi-tasks, which held out two time points (Supplementary Table S2).

### 3.2 PI-SDE reconstructs a biologically interpretable cellular potential energy landscape

We next trained PI-SDE using data from all time points and focused on the potential energy landscape learned by PI-SDE across two scRNA-seq datasets. Overall, the estimated potential energy values clearly recapitulated the developmental processes (Figs 2c and 3c). We hypothesized that the potential energy landscape derived by PI-SDE should be able to quantify cell differentiation potency. Specifically, we expected cells at earlier time points to exhibit higher potential energy when compared to more differentiated cells at later time points. Supplementary Fig. S2 confirmed this hypothesis, showing a clear trend of decreasing cell potential over time. To validate this observation statistically, we followed the testing approach proposed by Shi *et al.* (2019), and used the one-sided Wilcoxon rank-sum test between consecutive time points. Our results were highly significant, affirming that cells at earlier stages consistently had higher potential energy than those at later stages. For instance, in MH data, *P*-values were less than 2.95e-248 and 7.52e-62 when comparing Day 2–Day 4 and Day 4–Day 6, respectively.

For Veres data, the potential energy landscape reconstructed by PI-SDE exhibited progressive decline trend over developmental time (right panel of Fig. 2c). This trend was consistent with the underlying cellular dynamics. In contrast, the potential landscape reconstructed by PRESCIENT showed a sharp drop of potential energy for the cells collected at the final time point (Day 7) (middle panel of Fig. 2c). Moreover, this landscape by PRESCIENT seemed to be overly dominated by the data collected on Day 7, as shown by the red circled region in the left and middle panels of Fig. 2c. This phenomenon was likely due to the entropic regularization used by PRESCIENT, which aims to minimize the potential energy function at the final time point, possibly leading to an overfitting of the potential energy for cells at this stage. In addition, in the PRESCIENT’s landscape, we found that a handful of cells with high potential energy were scattered around the low potential energy area (the red circled region in the middle panel of Fig. 2c).

To further illustrate the superiority of our potential landscape, we visualized cellular velocity derived from the negative gradient of the resulting potential function. Since TrajectoryNet can directly infer cellular velocity, we added TrajectoryNet to the comparative analysis here (Fig. 2d). The cellular velocity predicted by PI-SDE correctly oriented cells toward terminal fates, whereas the vector fields inferred by both TrajectoryNet and PRESCIENT occasionally showed cells deviating from the data manifolds (e.g. the regions circled in red dashed lines in Fig. 2d). PI-SDE also successfully recapitulated the two differentiation branches during pancreatic *β*-cell differentiation, as shown in Fig. 2a. Specifically, for branch 1, our results clearly showed distinct directional differences in prog_sox2 and prog_nkx61 at Day 0, which stands in contrast to PRESCIENT’s results which indicated a homogeneous pattern, while TrajectoryNet displayed chaotic vectors.

As for MH data, we compared the potential energy and cellular velocity generated by PI-SDE, PRESCIENT (with growth rate), and PRESCIENT. In general, the estimated potential energy by PI-SDE clearly recapitulated the developmental process of mouse hematopoiesis, showing a transcriptional continuum from undifferentiated cells to mature cells. Similarly, the cellular velocity inferred by PI-SDE delineated the expected directional flow along the differentiation path when visualized on UMAP embedding (left panel of Fig. 3c).

By comparing the velocity for cells near the earliest time point (the regions circled in red dashed lines in Fig. 3c), we found that the cellular velocity inferred by PI-SDE clearly delineated the main cell lineages of monocyte and neutrophil. In contrast, the cellular velocity predicted by PRESCIENT (with growth rate) indicated a slightly trend of bifurcation, while PRESCIENT predicted chaotic movement in the early stages. As explored by PRESCIENT, the addition of cell growth rate altered the potential landscape near the earliest time point, improving the performance of fate prediction. We showed that our proposed physics-informed loss function could further improve the potential landscape near the earliest time point. This suggested that PI-SDE tended to capture cell fate information much earlier in time than PRESCIENT, even accounting for cell growth rate. To further demonstrate the superiority of our velocity, we followed the work of Yeo *et al.* (2021) to introduce *in silico* perturbations at the initial time point. Our results showed that PI-SDE was able to predict the expected outcome of transcription factor perturbations (Supplementary Fig. S3). This suggested that the cellular velocity generated by PI-SDE aligned well with the directional dynamics of cell movement (Supplementary Note S7).

### 3.3 HJ regularization stabilizes the training process

Next, we demonstrated the power of HJ regularization in the training process of PI-SDE. To illustrate its utility, we used the Veres data as an example and conducted a hyperparameter sensitivity analysis for the held-one-out task that removes data at Day 6. We set the diffusion coefficient as a vector parameter to be optimized during model training and explored a range of learning rates during optimization (lr = 0.001, 0.002, 0.005, and 0.01) and regularization strengths (*λ *= 0, 0.001, 0.01, 0.1, 0.5, and 1) to assess their impact on the model’s training and test performance.

As depicted in Supplementary Fig. S4, we observed that higher learning rates often led to instability, particularly when regularization strength *λ* did not exceed 0.01. For instance, when λ=0.001, an increase in the learning rate led to an increase in the best test loss for the held-out time (Day 6), from 42.880 (lr = 0.001) to 51.755 (lr = 0.01). However, the situation was reversed when λ>0.01. For example, when λ=0.05, the best test loss in held-out time (Day 6) decreased from 33.548 (lr = 0.001) to 27.377 (lr = 0.01), as learning rate increased. Since the magnitude of *λ* reflects the strength of HJ regularization in our physics-informed loss function, these results suggested that HJ regularization guided PI-SDE to find the optimal potential energy landscape with accurate predictions.

Beyond achieving superior test results, our comprehensive hyperparameter sensitivity analysis across a broad spectrum of parameter configurations revealed that PI-SDE achieved consistent and robust performance. As HJ regularization became more pronounced (*λ* ranging from 0 to 1), the model’s learning curves tended to converge and no longer fluctuated sharply (Supplementary Fig. S4). Such stability during the training process can be largely attributed to the integration of HJ regularization, since the physical principle, HJ equation, can be used to deduce that potential function is a Lyapunov function. A fundamental property of a Lyapunov function is its decreasing pattern over time, a characteristic that facilitates the analysis of global stability (Bressloff 2021). Consequently, the presence of HJ regularization (λ>0.01) significantly ensured that our learned potential function would satisfy HJ equation, thus contributing to a more stable convergence.

## 4 Discussion

In this paper, we propose PI-SDE, a physics-informed Neural-SDE framework, to reconstruct the underlying cellular potential energy landscape from time-series scRNA-seq data. PI-SDE extends the framework of PRESCIENT by leveraging the physical laws that govern the potential energy function–HJ equation. By integrating the HJ regularization, the resulting physics-informed loss function guides our model to accurately capture complex dynamics, while maintaining robustness and biological interpretability.

Generally, PI-SDE effectively combines theoretical principles with machine learning capabilities, thereby reducing overfitting and enabling the exploitation of large-scale, high-dimensional biological data. We have demonstrated the benefits of leveraging the physical insights.

First, PI-SDE maintained superior performance at unseen time points, especially on long-term prediction tasks. This implies that HJ regularization guides PI-SDE to a more accurate potential energy function space to faithfully recapitulate the underlying cellular dynamics.

Second, the potential energy landscape reconstructed by PI-SDE exhibited progressive decline trend over developmental time. The clear gradient trend highlights PI-SDE’s ability to capture the gradual evolution of cellular states from the undifferentiated states at higher energy levels to the differentiated states at lower energy levels. In contrast, PRESCIENT’s potential energy landscape appeared to be overfitted at the final time point on Veres data, resulting in cells at high and low potential being overlapped in gene expression space. This overlap could imply a lesser degree of change as detected by PRESCIENT, or a less sensitive capture of the dynamic cellular processes. Meanwhile, PI-SDE tended to capture cell fate information (e.g. trend toward bifurcation) in the earlier stage on both pancreatic *β*-cell differentiation and mouse hematopoiesis.

Third, solving SDEs is challenging owing to the inherent stochasticity. Therefore, careful design of the diffusion term is essential to improve stability and efficacy of modeling (Oh *et al.* 2024). Our results showed that the incorporation of HJ regularization can stabilize the training process, which is also theoretically substantiated. Overall, PI-SDE provides an effective and interpretable tool to model cellular dynamics for predicting gene expressions on unseen data and reconstruct the underlying potential energy landscape.

Nonetheless, some aspects still need to be improved. First, the current PI-SDE does not account for cell growth rate during cell development, possibly resulting in the assumption of conserved cell mass over time. Interestingly, the performance of PRESCIENT without growth rate was superior to PRESCIENT with growth rate, implying that the estimated growth rate may be inaccurate and thus inadvertently introducing bias into the model. We can solve this problem by adopting the unbalanced OT framework of Wasserstein–Fisher–Rao distance introduced by TIGON (Sha *et al.* 2024). Second, modeling of cell–cell communication in the SDE models can be challenging (Jiang *et al.* 2022), and we plan to pursue this topic in our future work.

## Supplementary Material

btae400_Supplementary_Data

## Data Availability

All the datasets used for analysis in this study are publicly available. For Veres data, we downloaded the data from GEO (GSE114412). For MH data, we obtained the raw data from the lineage_tracing folder in https://github.com/AllonKleinLab/paper-data. We provide a more detailed description of these datasets in the Supplementary Note S1.

## References

[btae400-B1] Bressloff PC. Stochastic Processes in Cell Biology. Vol. 41, 2nd edn. Heidelberg: Springer, 2021.

[btae400-B2] Fang X , Kruse K, Lu T et al Nonequilibrium physics in biology. Rev Mod Phys 2019;91:045004.

[btae400-B3] Goldstein H , Poole C, Safko J. Classical Mechanics. 3rd edn. New York: Pearson Education, 2011.

[btae400-B4] Huguet G , Magruder DS, Tong A et al Manifold interpolating optimal-transport flows for trajectory inference. Adv Neural Inf Process Syst 2022;35:29705–18. 37397786 PMC10312391

[btae400-B5] Jiang Q , Zhang S, Wan L. Dynamic inference of cell developmental complex energy landscape from time series single-cell transcriptomic data. PLoS Comput Biol 2022;18:e1009821. 35073331 10.1371/journal.pcbi.1009821 PMC8812873

[btae400-B6] Kidger P , Foster J, Li X et al Neural SDEs as infinite-dimensional GANs. In: *Proceedings of the 38th* *International Conference on Machine Learning, Vienna, Austria (virtual)*. PMLR, 2021, pp. 5453–63.

[btae400-B7] Li X , Wong T-KL, Chen RT et al Scalable gradients for stochastic differential equations. In: *Proceedings of the 23rd* *International Conference on Artificial Intelligence and Statistics, Palermo, Italy (virtual)*. PMLR, 2020, pp. 3870–82.

[btae400-B8] Oh Y , Lim D, Kim S. Stable neural stochastic differential equations in analyzing irregular time series data. In: *Proceedings of the 12th International Conference on Learning Representations*. Vienna, Austria, 2024.

[btae400-B9] Onken D , Fung SW, Li X et al OT-Flow: fast and accurate continuous normalizing flows via optimal transport. AAAI 2021;35:9223–32.

[btae400-B10] Ruthotto L , Osher SJ, Li W et al A machine learning framework for solving high-dimensional mean field game and mean field control problems. Proc Natl Acad Sci USA 2020;117:9183–93. 32273389 10.1073/pnas.1922204117 PMC7197015

[btae400-B11] Schiebinger G , Shu J, Tabaka M et al Optimal-transport analysis of single-cell gene expression identifies developmental trajectories in reprogramming. Cell 2019;176:928–43.e22. 30712874 10.1016/j.cell.2019.01.006 PMC6402800

[btae400-B12] Sha Y , Qiu Y, Zhou P et al Reconstructing growth and dynamic trajectories from single-cell transcriptomics data. Nat Mach Intell 2024;6:25–39. 38274364 10.1038/s42256-023-00763-w PMC10805654

[btae400-B13] Shi J , Li T, Chen L et al Quantifying pluripotency landscape of cell differentiation from scRNA-seq data by continuous birth-death process. PLoS Comput Biol 2019;15:e1007488. 31721764 10.1371/journal.pcbi.1007488 PMC6876891

[btae400-B14] Tong A , Huang J, Wolf G et al TrajectoryNet: a dynamic optimal transport network for modeling cellular dynamics. In: *Proceedings of the 37th International Conference on Machine Learning, Vienna, Austria (virtual)*. PMLR, 2020, pp. 9526–36. PMC8320749 34337419

[btae400-B15] Veres A , Faust AL, Bushnell HL et al Charting cellular identity during human in vitro *β*-cell differentiation. Nature 2019;569:368–73. 31068696 10.1038/s41586-019-1168-5 PMC6903417

[btae400-B16] Waddington CH. The Strategy of the Genes: A Discussion of Some Aspects of Theoretical Biology. London: G. Allen and Unwin, 1957.

[btae400-B17] Wang J. Landscape and flux theory of non-equilibrium dynamical systems with application to biology. Adv Phys 2015;64:1–137.

[btae400-B18] E Weinan , Li T, Vanden-Eijnden E. Applied Stochastic Analysis. Vol. 199. New York: American Mathematical Society, 2021.

[btae400-B19] Weinreb C , Rodriguez-Fraticelli A, Camargo FD et al Lineage tracing on transcriptional landscapes links state to fate during differentiation. Science 2020;367:eaaw3381. 31974159 10.1126/science.aaw3381 PMC7608074

[btae400-B20] Yang KD , Uhler C. Scalable unbalanced optimal transport using generative adversarial networks. In: *Proceedings of the 7th* *International Conference on Learning Representations. New Orleans, Louisiana,* 2019.

[btae400-B21] Yeo GHT , Saksena SD, Gifford DK. Generative modeling of single-cell time series with prescient enables prediction of cell trajectories with interventions. Nat Commun 2021;12:3222. 34050150 10.1038/s41467-021-23518-w PMC8163769

