# Supplementary material for: A physics-informed neural SDE network for learning cellular dynamics from time-series scRNA-seq data
Source: Bioinformatics. 2024 Sep 4;40(Suppl 2):ii120–7. doi: 10.1093/bioinformatics/btae400 (PMC11373338; doi:10.1093/bioinformatics/btae400)
Supplement: btae400_Supplementary_Data [file btae400_supplementary_data.zip › Jiang-249-SI.pdf]

A physics-informed neural SDE network for learning cellular dynamics from time-series scRNA-seq data

— Supplementary Information

Qi Jiang[1,2], Lin Wan[1,2,*]

[1] Academy of Mathematics and Systems Science, Chinese Academy of Sciences, Haidian District, 100190, Beijing, China

[2]School of Mathematical Sciences, University of Chinese Academy of Sciences, Beijing, China

[*]To whom correspondence may be addressed. Email: lwan@amss.ac.cn.

Supplementary Note

Supplementary Note S1: Datasets and preprocessing

For Veres data [1], we downloaded the data from GEO (GSE114412). The data comprised 51,274 cells, and the top 2,500 most variable genes had already been identified. Following the approach outlined by Yeo et al. [2], we used the Python package 'sklearn' to standardize the data and then exclude genes that had a correlation coefficient greater than 0.15 with TOP2A, as described by Veres et al. [1]. Subsequently, we computed a low-dimensional embedding using principal component analysis (PCA). The first 30 principal components (PCs) were used as input during training.

For MH data [3], we obtained the original data from https://github.com/AllonKleinLab/ paper-data/blob/master/Lineage_tracing_on_transcriptional_landscapes_links_state_to_fate_during _differentiation/README.md. The original data consists of 130,887 cells with measurements of 25,289 genes at 3 time points. Highly variable genes were identified following the methodology of Weinreb et al. [3], which involved filtering for variability and removing cell cycle-correlated genes. We then used the Python package 'sklearn' to perform data scaling and projected it into a low-dimensional space by PCA. Specifically, we selected the first 50 PCs as input for further analysis.

Supplementary Note S2: Details of Model implementation

In order to improve computational efficiency, we rewrite the mathematical form of $\mathrm{R_{HJ}}$ as

$$\begin{aligned}
\mathrm{R_{HJ}} &= \int_{t_0}^{t_T}\int_{\mathbb{R}^d}\left|\partial_t\Phi(\mathbf{x},t)-\frac{1}{2}\|\nabla_{\mathbf{x}}\Phi(\mathbf{x},t)\|^2\right|\rho_t(\mathbf{x})\mathrm{d}\mathbf{x}\mathrm{d}t &(1)\\
&= \int_{t_0}^{t_T}\int_{\mathbb{R}^d}\left|\partial_t\Phi\left(\mathbf{x}(\mathbf{x}_{t_0},t),t\right)-\frac{1}{2}\|\nabla_{\mathbf{x}}\Phi\left(\mathbf{x}(\mathbf{x}_{t_0},t),t\right)\|^2\right|\rho_t\left(\mathbf{x}(\mathbf{x}_{t_0},t)\right)\left|\frac{\partial\mathbf{x}(\mathbf{x}_{t_0},t)}{\partial\mathbf{x}_{t_0}}\right|\mathrm{d}\mathbf{x}_{t_0}\mathrm{d}t &(2)\\
&= \int_{t_0}^{t_T}\int_{\mathbb{R}^d}\left|\partial_t\Phi\left(\mathbf{x}(\mathbf{x}_{t_0},t),t\right)-\frac{1}{2}\|\nabla_{\mathbf{x}}\Phi\left(\mathbf{x}(\mathbf{x}_{t_0},t),t\right)\|^2\right|\rho_{t_0}\left(\mathbf{x}_{t_0}\right)\mathrm{d}\mathbf{x}_{t_0}\mathrm{d}t &(3)\\
&= \mathbb{E}_{\mathbf{x}_{t_0}\sim\rho_{t_0}}\int_{t_0}^{t_T}\left|\partial_t\Phi\left(\mathbf{x}(\mathbf{x}_{t_0},t),t\right)-\frac{1}{2}\|\nabla_{\mathbf{x}}\Phi\left(\mathbf{x}(\mathbf{x}_{t_0},t),t\right)\|^2\right|\mathrm{d}t. &(4)
\end{aligned}$$

where $\mathbf{x}(\mathbf{x}_{t_0},t)$ represents the state at time t, when x_{t_0} is the initial state at time t_0, ρ_{t_0} is the initial probability distribution of the modeling diffusion process $\{\rho(t) : t_0 \leq t \leq t_T\}$, i.e., $\hat{\rho}_{t_0}$.

Furthermore, the integration $\int_{t_0}^{t_T}\left|\partial_t\Phi\left(\mathbf{x}(\mathbf{x}_{t_0},t),t\right)-\frac{1}{2}\|\nabla_{\mathbf{x}}\Phi\left(\mathbf{x}(\mathbf{x}_{t_0},t),t\right)\|^2\right|\mathrm{d}t$ can be viewed as

a solution of the following differential function at time t_T, given an initial state $\mathbf{x}_{t_0}$,

$$\begin{aligned} \mathrm{d}r(\mathbf{x}_{t_0}, t) &= \left| \partial_t \Phi\left(\mathbf{x}(\mathbf{x}_{t_0}, t), t\right) - \frac{1}{2} \|\nabla_{\mathbf{x}} \Phi\left(\mathbf{x}(\mathbf{x}_{t_0}, t), t\right)\|^2 \right| \mathrm{d}t, \\ r(\mathbf{x}_{t_0}, t_0) &= 0. \end{aligned} \tag{5}$$

Therefore, we have

$$\mathrm{R}_{\mathrm{HJ}} = \mathbb{E}_{\mathbf{x}_{t_0} \sim \hat{\rho}_{t_0}} r(\mathbf{x}_{t_0}, t_T) \tag{6}$$

Then, the full optimization problem can be written as

$$\begin{aligned} &\min_{\{\Phi, \sigma\}} \mathcal{L}_{\mathrm{PI}} = \sum_{l=1}^{T} \mathrm{W}_2\left(\hat{\rho}_{t_l}, \rho_{t_l}\right)^2 + \lambda \mathbb{E}_{\mathbf{x}_{t_0} \sim \hat{\rho}_{t_0}} r(\mathbf{x}_{t_0}, t_T), \\ \text{s.t.} \quad &\partial_t \begin{pmatrix} \mathbf{x}(\mathbf{x}_{t_0}, t) \\ r(\mathbf{x}_{t_0}, t) \end{pmatrix} = \begin{pmatrix} -\nabla_{\mathbf{x}} \Phi(\mathbf{x}(\mathbf{x}_{t_0}, t), t)\mathrm{d}t + \sigma\left(\mathbf{x}(\mathbf{x}_{t_0}, t), t\right) \mathrm{d}W_t \\ \left|\partial_t \Phi(\mathbf{x}(\mathbf{x}_{t_0}, t), t) - \frac{1}{2}\|\nabla_{\mathbf{x}} \Phi(\mathbf{x}(\mathbf{x}_{t_0}, t), t)\|^2\right| \mathrm{d}t \end{pmatrix}, \\ &\begin{pmatrix} \mathbf{x}(\mathbf{x}_{t_0}, 0) \\ r(\mathbf{x}_{t_0}, 0) \end{pmatrix} = \begin{pmatrix} \mathbf{x}_{t_0} \\ 0 \end{pmatrix}, \\ &\mathbf{x}_{t_0} \sim \hat{\rho}_{t_0}, \quad \rho_{t_l} = \mathrm{Law}(\mathbf{x}_{t_l}). \end{aligned} \tag{7}$$

where $\mathrm{Law}(\mathbf{x}_{t_l})$ represents the predicted probability distribution of $\mathbf{x}_{t_l}$ by the SDE given $\{\Phi, \sigma\}$.

This form allows us to compute the states of $\mathbf{x}(\mathbf{x}_{t_0}, t)$ and $r(\mathbf{x}_{t_0}, t)$ for future times in parallel during the training process, thus significantly reducing computation time.

Supplementary Note S3: Baseline Models

We compared PI-SDE with the following three baseline methods.

- TrajectoryNet [4]: Tong et al. combine dynamic OT and continuous normalized flows (CNFs) to learn the optimal flow of the evolving cell populations in continuous time. Then, TrajectoryNet efficiently solves the transport problem using a Neural ODE framework. It is worth noting that TrajectoryNet offers the option to include 4 types of regularization. In TrajectoryNet's ablation study, the addition of density-based regularization and velocity regularization can have better and more robust results. However, estimated RNA-velocity is not available in general cases. Therefore, we only considered density-based regularization in the training of all datasets. We used Python codes on Github (https://github.com/KrishnaswamyLab/Trajectory-Net) to run TrajectoryNet.

- PRESCIENT [2]: Yeo et al. propose PRESCIENT (Potential eneRgy undErlying Single Cell gradIENTs), a generative modeling framework that models complex potential landscapes from time-series scRNA-seq data. PRESCIENT uses a neural network to decode

the underlying landscape of cell development. Notably, PRESCIENT can choose whether or not to include cell growth estimates in the modelling. For Veres data and MH data, which have already been studied by PRESCIENT, the growth rate information is available. Therefore, we trained both versions of PRESCIENT, with or without considering growth rate, for comparison. Throughout the paper, unless specified otherwise, "PRESCIENT" refers to the version without growth rate consideration. We used Python codes on Github (https://github.com/gifford-lab/prescient-analysis) to run PRESCIENT.

- MIOFlow [5]: Huguet et al. propose Manifold Interpolation Optimal-transport Flow (MIOFlow) to learn stochastic, continuous population dynamics from static snapshot data. MIOFlow combines dynamic models and optimal transport by training neural ordinary differential equations (Neural ODE) to continuously interpolate between static population snapshots. Additionally, MIOFlow integrates a geodesic autoencoder to further extend dynamic optimal transport into latent space. To be comparable with other methods, we directly feed the low-dimensional data (after PCA) into MIOFlow, i.e., set use_gae = False. We used Python codes on Github (https://github.com/KrishnaswamyLab/MIOFlow) to run MIOFlow.

Supplementary Note S4: Hyperparameter

For TrajectoryNet, we did a grid search over the strength of density-based regularization $\lambda_{\text{density}} \in \{0, 1, 2, 5, 10\}$ and learning rate $\text{lr} \in \{0.0001, 0.0005, 0.001\}$. All other hyperparameters retained TrajectoryNet's default parameters.

For PRESCIENT, we directly used the default hyperparameters for Veres data and MH data. For Veres data, the diffusion coefficient was set to 0.1, the strength of empirical regularization τ was set to 1e-6 and learning rate to 0.001. For MH data, the diffusion coefficient was set to 0.1, the strength of empirical regularization τ was set to 1e-6 and learning rate to 0.005. All other hyperparameters retained PRESCIENT's default parameters.

For MIOFlow, we did a grid search over learning rate $\text{lr} \in \{0.0001, 0.0002, 0.0005, 0.001\}$. All other hyperparameters retained MIOFlow's default parameters.

For PI-SDE, the potential function's architecture employed a fully connected 2-layer, 400-unit model with softplus activation, and the diffusion coefficient was set to a constant in all datasets. For both Veres data and MH data, the diffusion coefficient was set to 0.1, the strength of HJ regularization λ was set to 0.5 and the learning rate was set to 0.005. During training, gradient clipping was enforced with a maximum norm of 0.1. The optimization of the potential energy function was conducted using the Adam optimizer with batch size equivalent to one-tenth of the training set size. The Wasserstein distance computation employed the Sinkhorn algorithm [6] with

a scaling of 0.7 and a blur of 0.1.

Supplementary Note S5: Evaluation

We used the Wasserstein distance to measure performance of the model in predicting gene expression at testing time point, i.e., the difference between true and predicted data.

- Wasserstein distance, also known as the earth mover's distance or optimal transport distance, is a metric used to measure the dissimilarity between two probability distributions. It considers the optimal way to transport mass from one distribution to another and quantifies the minimum cost required to transform one distribution into another, where the cost is typically defined as the distance between points in a metric space. The Wasserstein distance between two probability distributions μ and ν defined on a metric space (X, d) is given by

  $$W_p(\mu, \nu) = \left(\inf_{\gamma \in \Gamma(\mu,\nu)} \int_{X \times X} d(x, y)^p d\gamma(x, y) \right)^{\frac{1}{p}},$$

  where $\Gamma(\mu, \nu)$ represents the set of all joint distributions whose marginals are μ and ν, and p is a parameter that determines the order of Wasserstein distance.

During training, both PI-SDE and PRESCIENT utilized the Sinkhorn algorithm with a scaling of 0.7 and a blur of 0.1 to calculate Wasserstein loss, since it is faster and more scalable. However, this approach yielded an entropy-regularized approximation rather than the exact value of Wasserstein distance. TrajectoryNet and MIOFlow employed the dynamic version of Wasserstein distance [7]. To ensure an unbiased and accurate evaluation, we used the 'emd2' function from the Python Optimal Transport ('pot') library to compute the exact Wasserstein loss. This metric provided a more precise measure of the model's generalizability.

Supplementary Note S6: Held-out tasks

To evaluate PI-SDE's performance in predicting gene expressions at unseen time points, we conducted held-out tasks on Veres data: (1) held-one-out task, where data from one time point was excluded during training, and (2) held-multi-out task, where data from multiple time points was excluded during training. After training on the remaining data, we assessed the trained model's ability to recover gene expression at the omitted (i.e., unseen) time points.

For Veres data, which includes data from 8 time points, we defined 10 sub-problems for analysis: (1) 7 held-one-out tasks, where each sub-problem used the complete data, except for the exclusion of one specific time point (other than the initial time point), (2) 3 held-multi-out tasks, where {Day 3, Day 4}, {Day 6, Day 7} and {Day 4, Day 7} were excluded, respectively.

To assess the model's ability to recover the unseen time point, we followed PRESCIENT's methodology. Initially, 10,000 cells were sampled at the initial time point. Subsequently, the model simulated the trajectory for each sampled cell until the evaluation time point. Finally, we computed the evaluation metric comparing the simulated cell population with the empirically observed cell population.

Supplementary Note S7: In silico perturbations

To test whether PI-SDE could exhibit the expected distributions of fates to biological systems under perturbed conditions, we followed the work of Yeo et al. [2] to introduce in silico perturbations, especially the perturbation of a specific transcription factor (TF). Perturbations were induced in silico by adjusting the scaled normalized expression of the target gene to z-score values below 0 for knockdowns (-0.5, -2, -2.5, and -5) and above 0 for overexpression (2, 5, and 10) at the initial time point. The perturbed gene expression profile was then reduced to lower-dimensional space through PCA for input into the forward simulation of the model that had already been trained on all time points.

In this study, we conducted in silico perturbation in the MH data, and chose TFs known to be involved in the regulation of monocyte (Irf8, Klf4) or neutrophil (Cebpe, Dach1) differentiation. As a control, we also tested two randomly selected non-TFs, including Dhrs2 and Sgk1. For each TF, we first randomly sampled 200 cells at the start time point (day 2) over 10 seeds. Then, we introduced different levels of gene overexpression or knockdown to the 200 cells at day 2 for each seed round. After reducing the perturbed gene expression profiles into 50-dimensional space using PCA, we used the trained PI-SDE to predict their distribution at the final moment (day 6). Ultimately, the distributions of the unperturbed cell population and the perturbed cell population at day 6 were compared.

We focused on the proportion of monocytes or neutrophils at day 6, as these TFs are involved in their regulation. To determine the cell type at the final time point, we used a voting classifier integrating Random Forest, k-Nearest Neighbors and Multi-Layer Perceptron classifiers. After splitting the data into training and test sets, each classifier was trained individually on the training data to classify cells as neutrophil, monocyte or other. The voting classifier then combined their predictions using a soft voting strategy. Additionally, the voting classifier was retrained on the entire data to improve its predictive performance. We then used the trained voting classifier to classify cells at day 6 and obtained the monocyte or neutrophil fractions for each TF in each seed round.

Overall, our results showed that PI-SDE was capable of predicting the expected outcomes of transcription factor perturbations (Fig. S3). As expected, we observed that up-regulation of TFs

that involved in monocyte development (Irf8 and Klf4) led to an increase in the fraction of monocyte cells compared to the baseline population, while down-regulation of these TFs resulted in a relative decrease in the fraction of monocytes (Fig. S3(a)). For TFs involved in neutrophil development, Cebpe and Dach1, we observed similar results (Fig. S3(b)). As for those non-TFs, Dhrs2 and Sgk1, These controls had negligible impact on the fraction of neutrophil and monocyte cells at day 6 (Fig. S3(c)). This outcome reinforced the robustness of our model against random variations.

Supplementary Tables

Table S1: Computational time of PI-SDE and other baseline methods.

	PI-SDE	PRESCIENT	TrajectoryNet	MIOFlow	# Timepoints	# Cells
Veres	80 minutes	24 minutes	148 minutes	3 minutes	8	51,274
MH	17 minutes	18 minutes	179 minutes	58 seconds	3	49,302

Table S2: Held-multi-out Performance across 5 seeds on Veres Data.

	held-out ts={3, 4}		held-out ts={6, 7}		held-out ts={4, 7}	
Model	train	test	train	test	train	test
TrajectoryNet	12.37 ± 0.84	10.78 ± 0.58	10.76 ± 0.97	14.86 ± 1.44	11.16 ± 0.96	12.15 ± 1.58
MIOFlow	10.56 ± 0.28	10.04 ± 0.31	10.40 ± 0.58	11.03 ± 0.21	10.41 ± 0.52	10.62 ± 0.25
PRESCIENT(+g)[1]	10.54 ± 1.02	8.72 ± 0.57	9.56 ± 0.77	14.64 ± 1.18	9.71 ± 1.09	11.00 ± 1.90
PRESCIENT	8.38 ± 0.25	7.90 ± 0.12	7.91 ± 0.37	10.29 ± 0.73	8.05 ± 0.31	8.32 ± 0.80
**PI-SDE**	$\mathbf{7.71 \pm 0.40}$	$\mathbf{7.75 \pm 0.22}$	$\mathbf{7.40 \pm 0.25}$	$\mathbf{9.62 \pm 0.86}$	$\mathbf{7.56 \pm 0.35}$	$\mathbf{7.98 \pm 0.75}$

The table presents results from two multi-time point held-out tasks where {Day 3, Day 4}, {Day 6, Day 7} and {Day 4, Day 7} were excluded from the training process, respectively. We compute the average Wasserstein loss between observed data and predicted data (training loss) and Wasserstein loss between unseen data predicted data (test loss).

[1] PRESCIENT with estimated growth rate.

Supplementary Figures

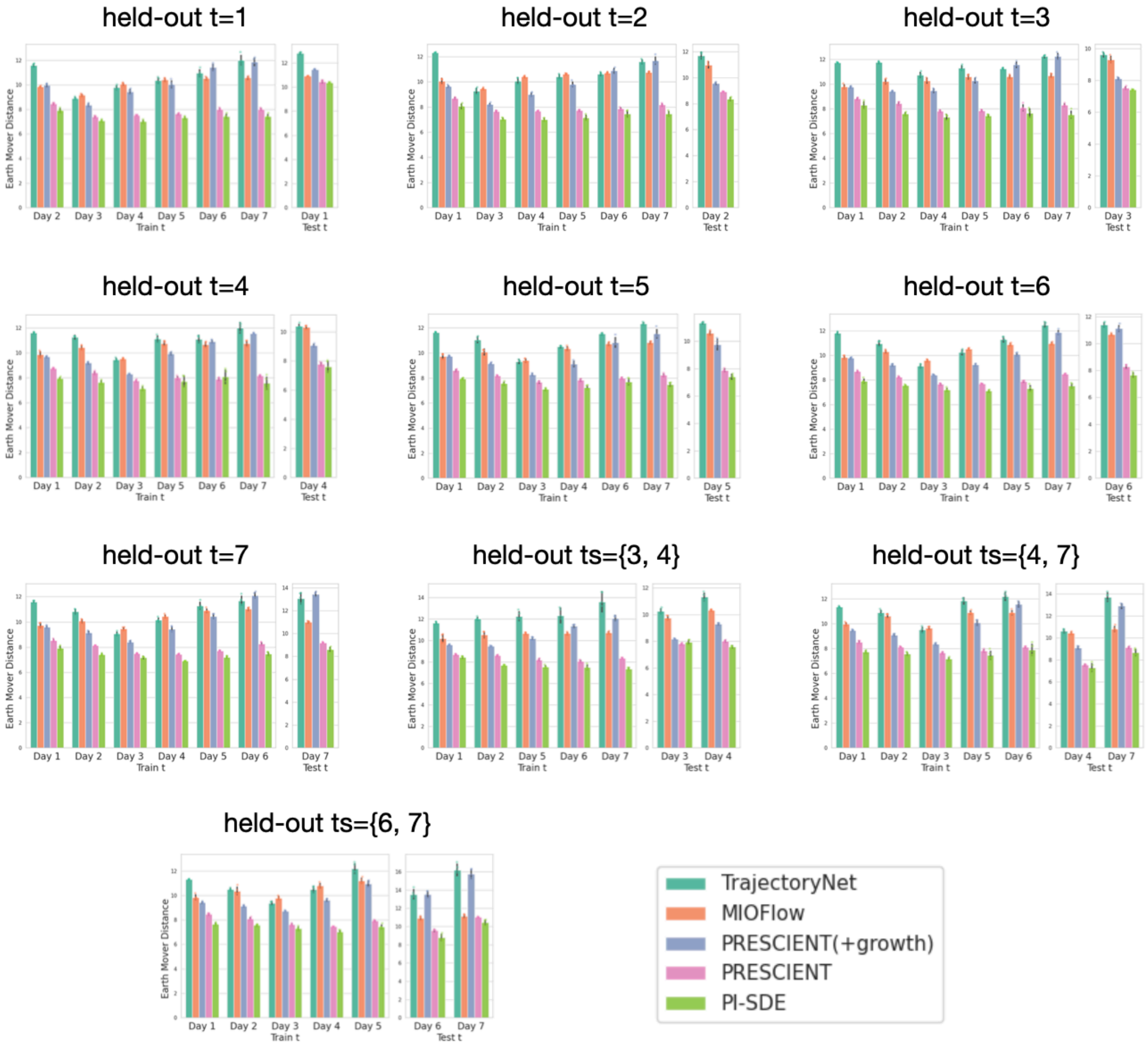

Fig. S1. Held-out Performance on Veres data.

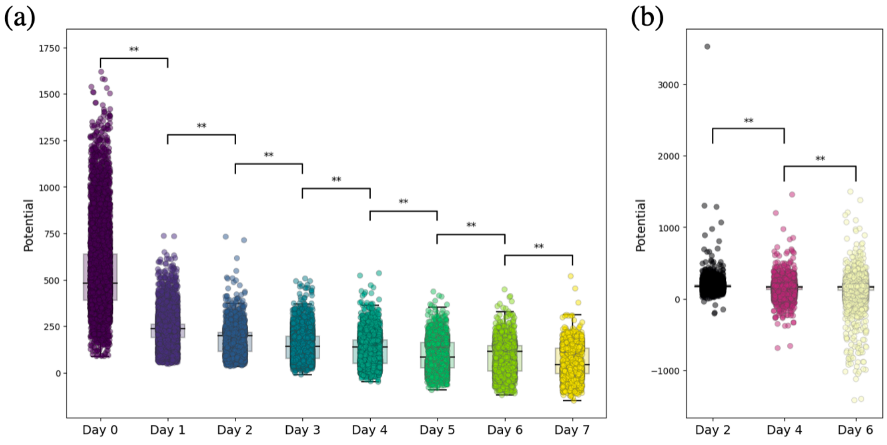

Fig. S2. Boxplot of potential energies of cells sampled at different time points for Veres data (a) and MH data (b). One-sided Wilcoxon ranksum statistic tests were conducted at two adjacent times. $**$ indicates a P-value smaller than 1e-60 by Wilcoxon test.

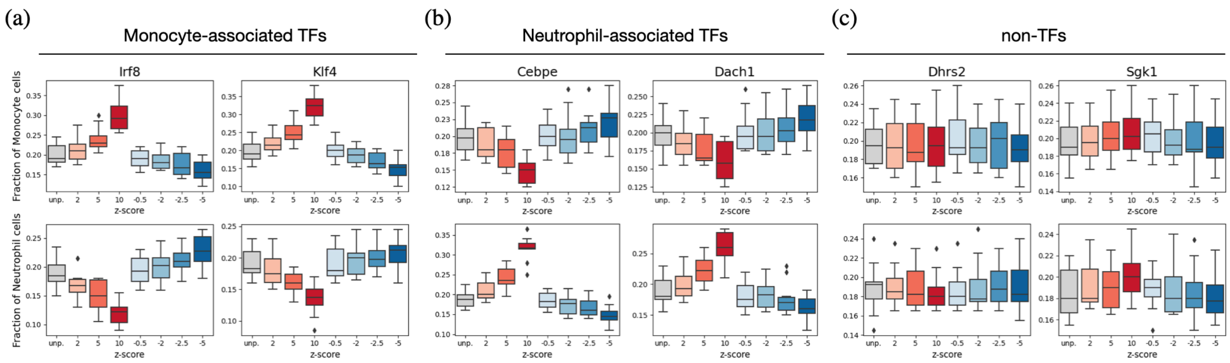

Fig. S3. Fraction of monocyte and neutrophil cells at final time point (day 6) over varying Z-score magnitudes of monocyte-associated TFs perturbation (a), neutrophil-associated TFs perturbation (b), and non-TF control gene perturbations (c). All boxplots are of randomly initialized unperturbed (unp.) vs. perturbed simulations (n = 10) with 200 cells for each initialization.

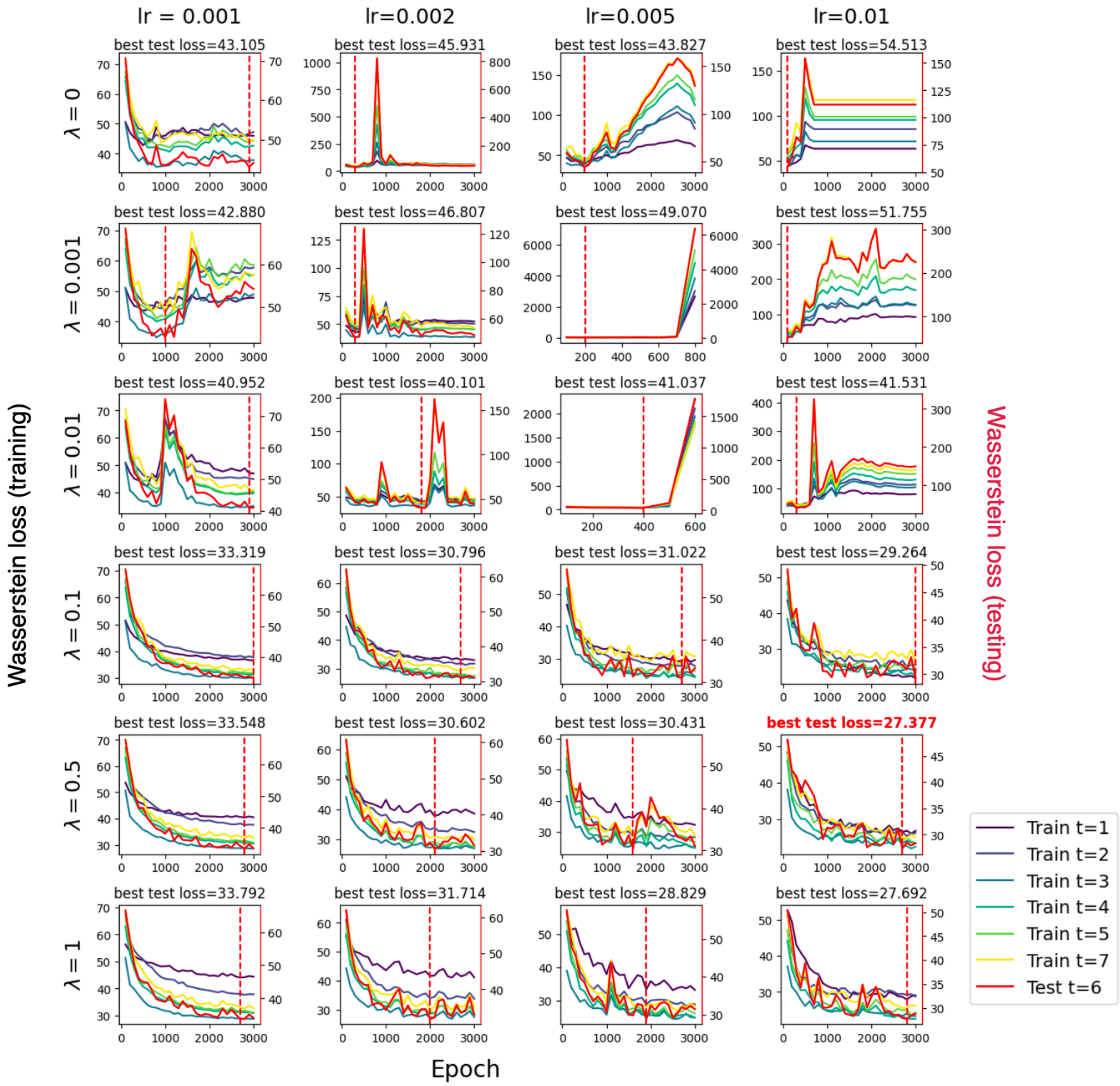

Fig. S4. Training dynamics of Wasserstein loss for training data and testing data over epoch on Veres data. This matrix of plots illustrates a comprehensive hyperparameter sensitivity analysis conducted to evaluate the effect of different learning rates (lr) and regularization parameters (λ) on the model's convergence behavior. Each subplot represents a unique combination of learning rate and regularization strength with the x-axis showing the number of epochs and the y-axis depicting Wasserstein loss.

Supplementary References

[1] Adrian Veres, Aubrey L Faust, Henry L Bushnell, Elise N Engquist, Jennifer Hyoje-Ryu Kenty, George Harb, Yeh-Chuin Poh, Elad Sintov, Mads Gürtler, Felicia W Pagliuca, et al. Charting cellular identity during human in vitro β-cell differentiation. *Nature*, 569(7756):368–373, 2019.

[2] Grace Hui Ting Yeo, Sachit D. Saksena, and David Kenneth Gifford. Generative modeling of single-cell time series with prescient enables prediction of cell trajectories with interventions. *Nature Communications*, 12, 2021.

[3] Caleb Weinreb, Alejo Rodriguez-Fraticelli, Fernando D Camargo, and Allon M Klein. Lineage tracing on transcriptional landscapes links state to fate during differentiation. *Science*, 367(6479):eaaw3381, 2020.

[4] Alexander Tong, Jessie Huang, Guy Wolf, David van Dijk, and Smita Krishnaswamy. Trajectorynet: A dynamic optimal transport network for modeling cellular dynamics. *Proceedings of machine learning research*, 119:9526–9536, 2020.

[5] Guillaume Huguet, Daniel Sumner Magruder, Alexander Tong, Oluwadamilola Fasina, Manik Kuchroo, Guy Wolf, and Smita Krishnaswamy. Manifold interpolating optimal-transport flows for trajectory inference. *Advances in Neural Information Processing Systems*, 35:29705–29718, 2022.

[6] Marco Cuturi. Sinkhorn distances: Lightspeed computation of optimal transport. *Advances in neural information processing systems*, 26, 2013.

[7] Jean-David Benamou and Yann Brenier. A computational fluid mechanics solution to the monge-kantorovich mass transfer problem. *Numerische Mathematik*, 84(3):375–393, 2000.
