# Supplementary material for: A physics-informed neural SDE network for learning cellular dynamics from time-series scRNA-seq data
Source: Bioinformatics. 2024 Sep 4;40(Suppl 2):ii120–7. doi: 10.1093/bioinformatics/btae400 (PMC11373338; doi:10.1093/bioinformatics/btae400)
Supplement: btae400_Supplementary_Data [file btae400_supplementary_data.zip › Jiang-249-lett.docx]

May 26, 2024

**Re: ECCB 2024 notification for paper 249 - conditionally accepted**

Dear ECCB2024 Organising committee,

We are pleased to submit our revised manuscript “A physics-informed neural SDE network for learning cellular dynamics from time-series scRNA-seq data” (paper-number: 249) for consideration for *ECCB 2024 Proceedings*.

We are very grateful to the referees' insightful comments and suggestions. They are very helpful for us to improve the research and to revise the manuscript. The manuscript has been fully revised by following the reviewers’ comments and suggestions, making our results more solid and convincing. We summarize the major changes as follows.

1) To clearly state the mathematical and physical foundations of our method, as suggested by Reviewer #1, we added a detailed description of the conceptual framework of quasipotential in the fifth paragraph of the Introduction (page 2 of the main text).
2) As suggested by Reviewer #2, we provided more held-out tasks, where multiple time points were excluded during training (Table S2, page 9 of Supplementary text).
3) As suggested by Reviewer #2, we included one more method, MIOFlow, for comparison in held-out tasks (Table 1, Table 2, page 5 of the main text; Table S2, Fig. S1, page 9-10 of Supplementary text).
4) As suggested by Reviewer #2, we added the explanation of “Law(xtl )” in Eq. 8 (page 4 of main text).
5) Besides, we revised the figures, and made many minor changes to improve the presentation.

We prepared the point-to-point response letters to each reviewer. We wish that these revisions were satisfactory. If there is any problem with the manuscript or files, please let us know.

Sincerely yours,

Qi Jiang and Lin Wan

Chinese Academy of Sciences

Responses to Reviewers:

We would like to thank the reviewers for thoughtful comments and valuable suggestions on our manuscript. The manuscript has been fully revised by following the reviewers’ comments and suggestions, making our results more solid and convincing.

The point‐by‐point responses to the reviewers’ comments and suggestions are as follows and our responses are in blue color.

Reviewer: 1

Comments to the Author

General comments:

This manuscript contains a high-quality presentation of an incremental advance to a computational technology addressing a genuine need. Single-cell expression measurement technology allows simultaneous capture of cell state from populations of cells where state is represented by gene expression profiles. A natural goal for time series of such data is inference of expression dynamics (and derivative characterizations such as differentiation/development state). However, because the data acquisition process is destructive to the cells, each expression profile in a time series never contains repeated measures of the same cells. Each time point profile contains measures of different cells, presumably subsets of a single changing population, with all the statistical trouble that entails. The manuscript builds on the strong foundations of earlier work, in particular PRESCIENT and Waddington-OT, and introduces an incremental advance in the form of a "physics-informed" regularization term, the Hamilton-Jacobi (HJ) equation from mechanics, to improve the learning of a potential function that putatively underlies the expression dynamics. The resulting improvements over its predecessors' implementations by various criteria (e.g. prediction accuracy, computational efficiency, etc.) were also incremental but substantial. In particular, the use of the HJ equation as a regularization term is a valuable idea which deserves publication with one caveat:

its application here is analogical and should be more clearly articulated as such lest readers are induced to conflate a useful model with reality. The Hamilton-Jacobi equation applies to dynamics of systems governed by conservation laws. No conserved quantity underlying developmental state has, to my knowledge, been articulated that would motivate a genuine potential function in the strictly-defined physics sense. The "quasi-potential" function usually associated with Waddington's epigenetic landscape metaphor is just that: quasi. I believe the authors are probably aware of this and would just recommend that the paper more clearly state that the existence of a potential function (even quasi-) is essentially a conjecture, the assumed truth of which leads to useful modeling formalisms and the applicability of the HJ equation. A troubling detail related to the preceding is the observation (between page 4 and 5) that the map yielded by PI-SDE is "smoother." One would hope it is smoother, and this should not be (written as if it were) a surprise. This is implicit in its being the gradient of a potential which the HG penalty is essentially forcing. It would indeed be very surprising if the resulting map was not smoother than e.g. PRESCIENT's. Alternatively, it is possible that these concerns are, in fact, addressed by the page 2 claim: "The potential energy function, also called the quasipotential, has been proven to satisfy the Hamilton-Jacobi (HJ) equation [Bressloﬀ, 2014]." I am doubtful that my concerns are *fully* addressed by that reference, but time does not permit exhaustive verification. If the authors believe my concerns are unfounded, a couple sentences elaborating the result from Bressloff 2014 would improve the theoretical basis of the manuscript and make it slightly more self-contained, which is always appreciated by readers. In any case, I believe the theoretical idea is valuable enough, in spite of the above concerns to support publication.

Reply: Thank you for your insightful comments. We agree with you that the application here is analogical. We have revised manuscript accordingly as follows.

(1) We pointed out that our proposed method is a conceptual framework, based on the energy landscape theory, that aims to learn the underlying potential energy landscape from time-series scRNA-seq data.

“PI-SDE extends the SDE-based framework of the PRESCIENT method by incorporating the principle of least action. The principle of least action is a fundamental concept in both physics and biology, which states that the optimal path taken by a system between two states is the one for which the action is minimized [Goldstein, 2011, Fang et al., 2019]. Recent advances of energy landscape theory provide a conceptual framework for analyzing the global stability and transition dynamics in biological systems [Wang, 2015]. In particular, the defined potential energy function, also called the quasipotential, becomes a powerful analytical tool for calculating the minimum action path of the noise-induced transition in multistable systems [Wang, 2015, Bressloff, 2021].” (the fifth paragraph in Introduction, page 2 of the main text).

(2) We revised the description of the potential energy landscape generated by PI-SDE as follows:

“For Veres data, the potential energy landscape reconstructed by PI-SDE exhibited progressive decline trend over developmental time (right panel of Fig. 2(c)). This trend is consistent with the underlying cellular dynamics. In contrast, the potential landscape reconstructed by PRESCIENT showed a sharp drop of potential energy for the cells collected at the final time point (Day 7) (middle panel of Fig. 2(c)).” (the second paragraph of section “PI-SDE reconstructs a biologically interpretable cellular potential energy landscape” in Results, page 4-5 of the main text)

(3) We specified the detailed conditions under which the claim on page 2 holds true:

“Large deviation theory provides a rigorous foundation that the quasipotential satisfies the Hamilton-Jacobi (HJ) equation within the context of SDEs with weak noise [Bressloff, 2021, Weinan et al., 2021]. The HJ equation motivates an important characterization for the least action path.” (the fifth paragraph in Introduction, page 2 of the main text).

Reviewer: 2

Comments to the Author

General comments:

Jiang et al. developed a neural network called PI-SDE to model time-series scRNA-seq data. It uses neural ODE to describe the dynamical changes of the gene expressions. The ODE has two components: one drift component is constrained by HJ equation, which always have a negative gradient, i.e. monotonically decreasing function; one fixed diffusion term governed by Brownian motion. The key contribution is the introduction of the HJ regularisation. The authors show that PI-SDE can predict the gene expression at an unknown time point with a better performance on one dataset, compared with PRESCIENT and TrajectoryNet. They also show that PI-SDE generate more gradual change in the differentiation path compared with PRESCIENT. In general, this is a nice illustration of the HJ regularisation in modelling the dynamics of gene expression. Pros 1. Interpretability of the HJ regularisation 2. The modelling itself.

Cons

1. Leave one time point out may not challenging enough.

Reply: Thank you for the constructive suggestion. In the revised manuscript, we added three held-multi-out tasks, where data from multiple time points was excluded during training, for Veres data (Supplementary Note S6: Held-out tasks, page 5-6 in the Supplementary text).

We compared these held-multi-out performance of PI-SDE with other three baseline methods in terms of the Wasserstein distance between predicted and true gene expression at training and test time points (Table. S2, Fig. S1 in the Supplementary text, page 9-10 in Supplementary text).

“Additionally, PI-SDE clearly outperformed the baselines in three held-multi- tasks, which held out two time points (Table S2).” (the third paragraph of section “PI-SDE accurately predicts gene expressions at unseen time points” in Results, page 4 of the main text).

2. MIOFlow is mentioned not compared

Reply: Thank you for your valuable suggestion. In the revised manuscript, we provide the comparison results of MIOFlow for all held-out tasks on Veres data (Table 1, Table 2, page 5 of the main text; Table S2, Fig. S1, page 9-10 of Supplementary text). PI-SDE still outperformed MIOFlow in terms of the Wasserstein distance between predicted and true gene expression at training (observed) and test (excluded) time points.

Minor comment

1. It will be nice to explain what is Law(xt) in eq. 4.

Reply: Thank you for your valuable suggestion. In the revised manuscript, we added the explanation of Law(xtl ) as

“ Law(xtl ) represents the predicted probability distribution of xtl by the SDE given {Φ, σ}.” (page 4 of the main text)
